# Salivary gland-type cancers: cross-organ demographics of a rare cancer

**DOI:** 10.1007/s10147-024-02505-3

**Published:** 2024-03-16

**Authors:** Aika Tanzawa, Kengo Saito, Masayuki Ota, Koji Takahashi, Izumi Ohno, Toyoyuki Hanazawa, Katsuhiro Uzawa, Yuichi Takiguchi

**Affiliations:** 1 Department of Medical Oncology, Graduate School of Medicine, Chiba University, 1-8-1, Inohana, Chuo-ku, Chiba, 260-8670 Japan; 2 Department of Oral Science, Graduate School of Medicine, Chiba University, Chiba, Japan; 3 Department of Molecular Virology, Graduate School of Medicine, Chiba University, Chiba, Japan; 4 Department of Diagnostic Pathology, Graduate School of Medicine, Chiba University, Chiba, Japan; 5 Department of Otorhinolaryngology/Head and Neck Surgery, Graduate School of Medicine, Chiba University, Chiba, Japan

**Keywords:** Cross-organ, Broncho-pulmonary, Adenoid cystic carcinoma, Mucoepidermoid carcinoma, Epithelial-myoepithelial carcinoma, Acinic cell carcinoma

## Abstract

**Background:**

Salivary gland-type cancers (SGTCs) are histologically heterogeneous and can affect organs other than the salivary glands. Some tumors outside the salivary glands are diagnosed on their unique histological characteristics. Comprehensive cross-organ studies on SGTCs are limited.

**Methods:**

We retrospectively analyzed the data of patients with salivary duct carcinoma (SDC), adenoid cystic carcinoma (AdCC), mucoepidermoid carcinoma (MEC), epithelial-myoepithelial carcinoma (EMC), acinic cell carcinoma (AcCC), and polymorphous adenocarcinoma (PAC) who visited our institution between 2009 and 2019. The primary tumor sites were classified into four categories; major salivary glands, head/neck (H/N) excluding (exc) major salivary glands (MSG) regions, broncho-pulmonary regions, and “others”. H/N exc MSG was further divided into three subcategories, nasal/paranasal sinus, oral and pharynx/larynx.

**Results:**

We identified 173 patients with SGTCs, with SDC, AdCC, MEC, EMC, AcCC, and PAC accounting for 20%, 42%, 27%, 3%, 8%, and 1% of the cases, respectively. The most frequent primary site was the major salivary glands (64%), followed by H/N exc MSG regions (27%), broncho-pulmonary regions, and “others”, thus non-salivary gland origins accounted for 9% of all cases. Patients with SDC, MEC, AcCC, or SGTC of the major salivary glands and broncho-pulmonary regions were more frequently treated by surgery. The overall survival time of the patients with MEC was significantly better than that of patients with SDC or EMC.

**Conclusions:**

This cross-organ study highlights the clinical significance of SGTCs, underscoring the need for developing novel therapies for this rare disease entity.

## Introduction

Salivary gland tumors are rare, occurring at an incidence of 2.5 to 3.0 cases per 100,000 people per year in the Western world [1]. As of 2021, malignant salivary gland tumors accounted for only 0.2% of all cancers registered by the “Designated Cancer Care Hospitals” in Japan [2]. However, these tumors are heterogeneous, comprising almost 40 histological types [3]. Among these, carcinomas account for approximately 20% of all tumors of the parotid gland, 50% of all tumors of the submandibular glands, and 80% of all tumors of the minor salivary glands [4, 5]. Malignant tumors of the salivary glands include salivary duct carcinoma (SDC), adenoid cystic carcinoma (AdCC), mucoepidermoid carcinoma (MEC), epithelial-myoepithelial carcinoma (EMC), acinic cell carcinoma (AcCC), polymorphous adenocarcinoma (PAC), and others [6].

A majority of the aforementioned tumors are considered to originate from the three major salivary glands: the parotid, the submandibular, and the sublingual glands. However, they sometimes originate from the minor salivary glands [7], which are located throughout the aerodigestive tract. Besides salivary glands, these tumors can also rarely develop in a wide variety of other organs, including broncho-pulmonary sites, breast, skin, and other organs [8]. The World Health Organization defines such tumors as salivary gland-type tumors and lists AdCC, MEC, EMC, and other tumors including benign tumors, in broncho-pulmonary salivary gland-type tumors [9]. AcCC, AdCC, EMC, and other tumors, including benign tumors, are listed as salivary gland-type tumors of the breast [10]. Even when limited to malignant histological types, that is, salivary gland-type carcinomas (SGTCs), most such tumors arising in the lungs are considered as low-grade malignancies due to their slow-growing nature with limited capability for invasion and metastasis [11]. However, to the best of our knowledge, few studies have been conducted to compare the incidence, pathological subtype distribution, clinical stage, and prognosis of various organ-specific SGTCs, presumably because comprehensive investigations of SGTCs outside the salivary glands and lungs are scarce.

The treatment mainstay for SGTCs, including primary salivary gland cancers in the early stage is curative surgery [7, 12, 13], whereas no standard of care has been established for locally advanced or metastatic disease, even for the case of primary salivary gland cancers, suggesting as-yet unmet medical needs [14]. Molecular-targeted therapy has recently begun to be used for SGTCs, and druggable molecular targets in SDC specifically include the androgen receptor, *ErbB2/HER-2*, *BRAF*, tumor mutation burden [15], and *NTRK* gene-fusion, seen in ≥ 80% of salivary gland secretary carcinomas [16]. For further development of optimal therapies, more comprehensive epidemiological studies of SGTCs involving the salivary glands and other organs are essential, while most previous research was mainly organ specific. Among the various histological types of SGTCs, SDC, AdCC, MEC, EMC, AcCC and PAC have distinctive histopathological characteristics and can be relatively clearly classified as SGTCs, even when they occur at primary sites other than the salivary glands, in contrast to the case for other histological types such as adenocarcinoma not otherwise specified and squamous cell carcinoma. The present study was aimed as a cross-organ overview of SGTCs limited to SDC, AdCC, MEC, EMC, AcCC, and PAC, and to compare these subtypes of SGTCs arising in different organs, including organs other than the salivary glands, based on the experience at a single institution.

## Methods

### Patients

Patients with SGTCs, specifically SDC, AdCC, MEC, EMC, AcCC, or PAC in any organ were extracted from a database encompassing all patients who visited our institution for the first time between January 2009 and December 2019. The extraction process involved searching for the ICD-O codes 8500/3, 8200/3, 8430/3, 8562/3, 8550/3, 140/3, as well as searching using the keywords of SDC, AdCC, MEC, EMC, AcCC, and PAC. The end of the study period was set at December 2019 to allow for a sufficient follow-up time for analysis of the prognosis. Clinical records, including pathology reports and surgical records, of the extracted patients were meticulously reviewed individually to ensure the accuracy of the diagnosis.

### Study design, classification of the primary site, statistical methods and ethical considerations

The study subjects included patients with SGTCs affecting various organs, but limited to SDC, AdCC, MEC, EMC, AcCC, or PAC. All pathological diagnoses were based on histological evaluation of surgical or biopsy specimen, and patients diagnosed by cytology alone were not included in the study. The clinical parameters analyzed included the gender, age at the initial diagnosis of SGTC, smoking status, clinical/pathological presence of nodal involvement, distant metastasis, primary site of cancer, treatment methods adopted, and overall survival. The tumor primary sites were categorized into the following four groups: (1) major salivary glands comprising the parotid, submandibular and sublingual glands, (2) head/neck (H/N) excluding (exc) major salivary glands (MSG) regions, (3) broncho-pulmonary regions, and (4) “other” regions. H/N exc MSG regions included the nasal cavity, paranasal sinuses, palate, buccal mucosa, oral floor, tongue, gingiva, pharynx, and larynx, and was further divided into three subcategories, nasal/paranasal sinus, oral and pharynx/larynx. The incidence of each pathological type of SGTC was evaluated in the entire population and according to the primary site. Age comparisons among different groups were conducted using the Kruskal–Wallis test. Comparisons of the sex, smoking status, presence of nodal involvement, distant metastasis according to the histological type, distribution of histological types among primary sites, and difference of treatment according to the primary site and histological type were performed using Fisher’s exact test. Overall survival from diagnosis to death from any cause was determined by the Kaplan–Meier method and compared using the log-rank test. Multiple groups more than 2 groups were first compared among all groups together. When a statistical difference was found, individual comparisons between each pair were conducted to avoid the bias of multiplicity of statics. P < 0.05 (two sided) was set as denoting statistical significance. The statistical analyses were conducted using JMP Pro, ver. 15.0.0. The study protocol was reviewed and approved by the institutional review board of the Graduate School of Medicine, Chiba University (approval number M10019). The need to obtain patient written informed consent for participation this study was waived and an opt-out method was applied to obtain consent based on indication from the IRB, due to the retrospective nature of the study and absence of any intervention.

## Results

### Patient characteristics

**Figure 1:**
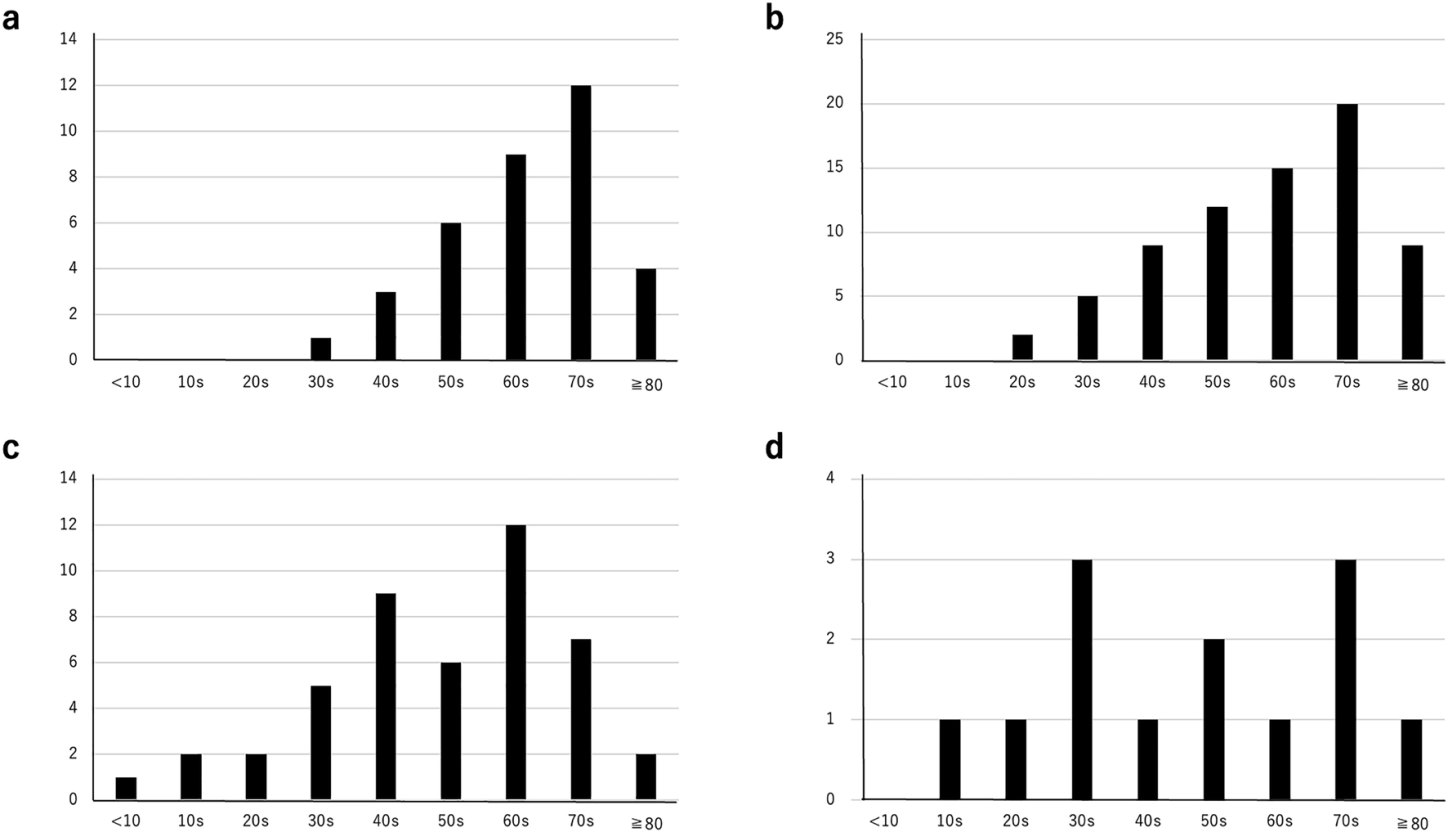
Age distribution of the patients with SDC (**a**), AdCC (**b**), MEC (**c**) and AcCC (**d**) are depicted. The horizontal axis represents the age groups, while the vertical axis indicates the number of patients. Patients with SDC and AdCC were predominantly in their 70s, whereas patients with MEC were predominantly in their 60s. Patients with AcCC distribute evenly throughout all age group without a peak

After extracting the patients through the database search using the ICD codes and key words, the records of individual patients were scrutinized to exclude patients with other diseases, patients in whom the diagnosis was not based on histological evaluation at our institution, and patients who were referred solely for a second opinion. Finally, 173 patients with SGTCs, including SDC, AdCC, MEC, EMC, AcCC, or PAC at any primary site were identified. Table 1 shows the characteristics of the patients. The cohort comprised 45% women, with a median age of 64 (range 6–92) years. Patients with SDC, AdCC, MEC, EMC, AcCC, and PAC accounted for 20%, 42%, 27%, 3%, 8%, and 1% of the entire cohort, respectively. There was statistically significant difference in the gender distribution according to the histological type where male was more predominant in SDC than AdCC and MEC. The patient age also differed significantly among the histological types (Fig. 1). In regard to the age distribution as a function of the histological type, the patients with SDC and AdCC were predominantly in their 70s, whereas patients with MEC were predominantly in their 60s. Patients with AcCC distributes evenly throughout all age group without a peak. The smoking status did not differ significantly among the histological types. The percentage of patients with nodal involvement/distal metastasis differed significantly among the histological types, with SDC patients having more positive N and M factors than other histological types (Table 1).

**Table 1:** Patient characteristics

Parameters	Overall	SDC	AdCC	MEC	EMC	AcCC	PAC	*p* value
	(n = 173)	(n = 35)	(n = 72)	(n = 46)	(n = 6)	(n = 13)	(n = 1)	
Sex, women, no (%)	78 (45.1)	5 (14.3)	37 (51.4)	28 (60.7)	2 (33.3)	4 (30.8)	1 (100)	< 0.0001*
Age, years, median (range)	64 (6–92)	68 (38–87)	65.5 (28–87)	54.5 (6–92)	74 (42–79)	57 (11–85)	80	0.0033**
Smoker, % (rate)***	39 (59/153)	38 (10/26)	42 (28/66)	27 (12/45)	100 (4/4)	42 (5/12)	0 (0/1)	0.0561*
Positive N factor, % (rate)***	25 (41/165)	65 (20/31)	14 (10/70)	20 (9/45)	17 (1/6)	1 (1/13)	0 (0/1)	< 0.0001*
Positive M factor, % (rate)***	3 (5/166)	13 (4/31)	6 (4/71)	0 (0/46)	17 (1/6)	0 (0/13)	0 (0/1)	0.0153*
Primary site, no (%)								
Major salivary glands	110 (63.6)	35 (100)	24 (33.3)	35 (76.1)	5 (83.3)	11 (84.6)	0	< 0.0001*
Head/neck exc MSG	47 (27.2)	0	37 (51.4)	6 (12.8)	1 (16.7)	2 (15.4)	1 (100)	
[Nasal/paranasal sinus]	[24 (13.9)]	[0]	[21 (29.2)]	[2 (4.3)]	[0]	[1 (7.7)]	[0]	
[Oral]	[16 (9.2)]	[0]	[11 (15.3)]	[3 (6.5)]	[1 (16.7)]	[0]	[1 (100)]	
[Pharynx/larynx]	[7 (4.0)]	[0]	[5 (6.9)]	[1 (2.2)]	[0]	[1 (7.7)]	[0]	
Broncho-pulmonary	10 (5.8)	0	6 (8.3)	4 (8.7)	0	0	0	
Others	3 (3.5)	0	5 (6.9)	1 (2.2)	0	0	0	

*Fisher’s exact test

**Kruskal–Wallis test

***The denominator is not equal to the total number due to missing data

### Distribution of the primary sites

The primary sites of the tumors in the 173 patients were the major salivary glands (n = 110), H/N exc MSG regions (n = 47), broncho-pulmonary regions (n = 10), and “others” (n = 6). The numbers of patients with each histological type in each of the major salivary glands and each of the H/N exc MSG regions are shown in Table 2. Specific information about patients classified as having tumors arising from sites included in the “others” category is presented in Table 3. Among the tumors arising from the major salivary glands, 70% (77/110) originated in the parotid gland, 24% (26/110) in the submandibular glands, and 6% (7/110) in the sublingual glands. In regard to the tumors arising from other H/N exc MSG regions, paranasal sinuses were the most frequently affected sites, followed by the nasal cavity, oral floor, and the nasopharynx (Table 2). In 6 patients, the SGTCs arose from sites included in the “others” category, including the orbit, lacrimal gland, Bartholin gland, and skin (Table 3).

**Table 2:** Number of patients according to the histological type and primary site (major salivary glands and other H/N regions)

	All patients	SDC	AdCC	MEC	EMC	AcCC	*p* value*
	(n = 110)	(n = 35)	(n = 24)	(n = 35)	(n = 5)	(n = 11)	
Major salivary glands							
Parotid	77	28	9	25	4	11	0.0025
Submandibular	26	7	12	6	1	0	
Sublingual	7	0	3	4	0	0	
H/N exc MSG regions	(n = 47)	(n = 0)	(n = 37)	(n = 6)	(n = 1)	(n = 2)	
Nasal/paranasal sinus							
Nasal cavity	8	0	7	1	0	0	0.0076
Paranasal sinuses	16	0	14**	1***	0	1***	
Oral							
Palate	4	0	3	0	0	0	
Buccal mucosa	4	0	2	2	0	0	
Oral floor	5	0	3	1	1	0	
Tongue	1	0	1	0	0	0	
Gingiva	2	0	2	0	0	0	
Pharynx/larynx							
Nasopharynx	5	0	3	1	0	1	
Hypopharynx	1	0	1	0	0	0	
Larynx	1	0	1	0	0	0	

*Fisher’s exact test

**All patients had tumors in the maxillary sinus

***Ethmoid sinus

**Table 3:** Specific information of 6 patients with SGTCs originated at other sites

Age	Sex	Primary site	Histological type	Treatment method
77	M	Orbit	MEC	RT and CT
75	M	Lacrimal gland	AdCC	No information^a^
87	F	Bartholin gland	AdCC	RT alone
45	M	Skin	AdCC	ST alone
81	F	Skin	AdCC	ST and CT
57	F	Skin	AdCC	ST and RT

ST, surgical treatment; RT, radiotherapy; CT, chemotherapy; BSC, best supportive care alone

^a^Data not obtained because the patient was referred to another institution for treatment.

### Characteristics of each histological type

The distribution of the histological type varied significantly among the primary sites. The most frequent primary sites of origin of SGTCs overall, SDC, MEC, EMC and AcCC were the major salivary glands, followed by H/N exc MSG regions. SDC was exclusively originated from major salivary glands. In contrast, the most frequent sites of origin of AdCCs were H/N exc MSG regions, especially nasal/paranasal sinus, followed by the major salivary glands (Table 1). The cohort included a single patient of PAC arising from the palate (Table 2). SGTCs originating from the broncho-pulmonary regions consisted of 6 patients with AdCC and 4 patients with MEC. Distribution of the histological types also differed significantly among the major salivary glands: 80% (28/35) of all SDC and 71% (25/35) of all MEC arose in the parotid gland, whereas 50% (12/24) of all AdCC arose in the submandibular gland. In all the cases of AcCC (n = 11) arising from the major salivary glands, the primary site was the parotid gland (Table 2).

### Therapeutic modalities and prognosis

**Figure 2:**
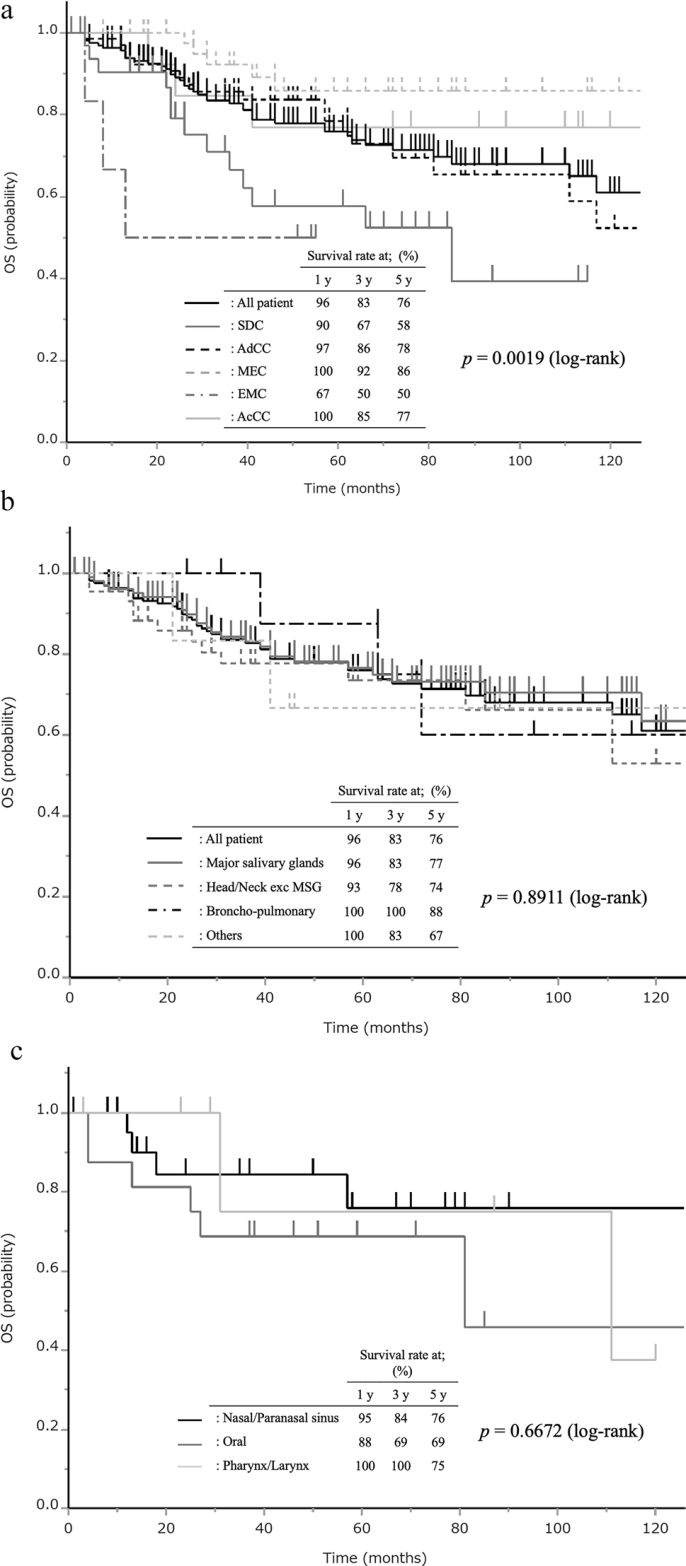
Overall survival curves are presented based on the histological type (**a**), and according to the primary site (**b**), together with overall survival curves of the three subcategories of H/N exc MSG (**c**). The curves for all patients are duplicated in (**a**) and (**b**). Vertical lines denote censored cases. The log-rank test was conducted to compare among all groups together

The treatment strategies adopted for the patients overall, and those according to the primary sites and histological types are presented in Table 4. Overall, 40% (70/173) of patients were treated by surgery alone, and an additional 33% (57/173) were treated by surgery in combination with other modalities (Table 4). In regard to the treatment method according to the primary site, surgical treatment alone or combined with other modalities was undertaken in 84% (92/110) of cases of SGTCs arising from the major salivary glands, 49% (23/47) of SGTCs arising from other H/N exc MSG regions, 90% (9/10) of SGTCs arising from the broncho-pulmonary regions, and 50% (3/6) of SGTCs arising from sites classified as “others.” In regard to the treatment according to the histological type, surgical treatment alone or combined with other modalities was undertaken in 91% (32/35) of cases of SDC, 57% (41/72) of cases of AdCC, 85% (39/46) of cases of MEC, 67% (4/6) cases of EMC, and 92% (12/13) of cases of AcCC. There were statistically significant differences in the therapeutic strategies adopted according to the primary site and histological type. More specifically, patients with tumors arising from the major salivary glands were more often treated by surgery than those with tumors arising from other H/N exc MSG regions. SDC, MEC and AcCC were more often treated by surgery than AdCC (Table 4). Consequently, the overall survival (OS) rates of all the patients with SGTCs were good, with 1-, 3-, and 5-year survival rates of 96%, 83%, and 76%, respectively. At the median follow up period of 50 months, 74% of the patients were alive, and the median OS was not yet reached (Figs. 2a). The OS tended to vary according to the histological type (p = 0.0019), and patients with MEC showed a better prognosis than those with SDC (p = 0.0007) or EMC (p = 0.0048), with 5-year OS rates of 86%, 58%, and 50%; and patients with AdCC showed a better prognosis than those with EMC (p = 0.010) with 5-year OS rates of 78% and 50%, respectively; the differences were statistically significant (Fig. 2a). As for prognosis according to the primary site, there was no statistically significant differences among the five categories (Fig. 2b). In addition, there was no statistically significant differences among the three subcategories of patients with SGTCs originated from H/N exc MSG regions (Fig. 2c).

**Table 4:** Treatment strategies adopted according to the primary site and histological type

	Primary site (p < 0.0001)*							Histological type (p = 0.0002)**					
	Major salivary glands	H/N exc MSG				Broncho- pulmonary	Others	SDC	AdCC	MEC	EMC	AcCC	PAC
		[Nasal/ paranasal sinus]	[Oral]	[Pharynx/ larynx]	Subtotal								
	(n = 110)	[(n = 24)]	[(n = 16)]	[(n = 7)]	(n = 47)	(n = 10)	(n = 6)	(n = 35)	(n = 72)	(n = 46)	(n = 6)	(n = 13)	(n = 1)
ST included, n (%)	92 (84)	[8 (33)]	[11 (69)]	[4 (57)]	23 (49)	9 (90)	3 (50)	32 (92)	41 (57)	39 (85)	4 (67)	12 (92)	1 (100)
ST alone	49	[3]	[7]	[4]	44	6	1	8	19	31	3	10	1
ST and RT	30	[4]	[1]	[0]	5	3	1	17	15	5	1	1	0
ST and CT	5	[0]	[2]	[0]	2	0	1	4	3	1	0	0	0
ST, RT and CT	8	[1]	[1]	[0]	2	0	0	3	4	2	0	1	0
ST excluded, n (%)	18 (16)	[16 (67)]	[5 (31)]	[3 (43)]	24 (51)	1 (10)	3 (50)	3 (9)	31 (43)	7 (15)	2 (33)	1 (8)	0
RT alone	4	[8]	[2]	[2]	12	0	1	0	16	2	0	0	0
CT alone	0	[0]	[1]	[0]	1	0	0	0	0	0	1	0	0
ICI	1	[0]	[1]	[0]	1	0	0	0	1	1	0	0	0
RT and CT	0	[3]	[0]	[0]	3	0	1	0	3	1	0	0	0
BSC	3	[0]	[0]	[0]	0	0	1	0	0	0	0	0	0
Referral^a^	10	[5]	[1]	[1]	7	1	1	3	11	3	1	1	0

ST, surgical treatment; RT, radiotherapy; CT, chemotherapy; ICI, immune checkpoint inhibitor; BSC, best supportive care alone

*Difference in the rate of adoption of surgery according to the primary site (Fisher’s exact test)

**Difference in the rate of adoption of surgery according to histological type (Fisher’s exact test)

^a^Referral to other institutions

## Discussion

In the present study, we investigated the cross-organ demographics of SGTCs based on data from a study cohort of 173 patients. In agreement with previous reports [1, 4, 5], the three major salivary grands were the most frequent primary sites of origin of SGTCs, however, these tumors arising in tissues outside the major salivary glands, most commonly from H/N exc MSG regions that consisted of nasal/paranasal sinus, oral and pharynx/larynx sub-regions, broncho-pulmonary regions, and “others,” accounted for as many as 36% of cases. SGTCs arising from other H/N exc MSG regions are considered most likely to originate from the minor salivary glands, although it would be very difficult to definitively distinguish SGTCs arising from the minor salivary glands from those arising from other tissues. Besides the major salivary glands and likely minor salivary glands, in 5.8% and 3.5% of the overall study population, the tumors originated from the broncho-pulmonary regions and “others”, respectively. Therefore, the actual frequency of rare SGTCs of most likely non-salivary gland origin was 9.3% in the present cohort. Nevertheless, the finding should be interpreted with caution, because the possibility of the tumors being metastatic tumors from unknown primary sites cannot be completely excluded. In addition to the demographics of SGTCs according to the primary sites, we also investigated the demographics of SGTCs according to the histological types in this study. The three major histological types of SGTCs, SDC, AdCC and MEC, accounted for 88% of all SGTCs, with SDC accounting for 20% (35/173), AdCC accounting for 42% (72/173) and MECs accounting for 27% (46/173) of all cases. However, there appeared to be some tendency towards variability of distribution of the histological types according to the primary sites; SDC (32%, 35/110) and MEC (32%, 35/110) seemed to be the predominant type in the major salivary glands, while AdCC seemed to be the predominant type (79%; 37/47) in other H/N exc MSG regions, and most likely of minor salivary gland origin. These findings appear to be consistent with previous reports [1, 4, 5]. Although the sample size was limited, we found from the present study, that AdCC is the predominant histological type among non-major salivary gland SGTCs. Besides all broncho-pulmonary SGTCs being AdCC (6/10) or MEC (4/10), 5 out of 6 patients with tumors arising from “others,” including 3 with the tumors arising from the skin, had AdCC. These findings appeared to differ from the finding in tumors arising from the major salivary glands.

Although also potentially influenced by the disease extent, SDC, MEC and AcCC were more frequently treated by surgery than AdCC and EMC. SGTCs originating from the major salivary glands were also more frequently treated by surgery as compared with SGTCs arising from other organs, and this could result in the better prognosis of patients with major salivary gland SGTCs than those with SGTCs arising from other sites. The prognosis of patients with broncho-pulmonary SGTCs was also good, with a 5-year OS rate of 88%, possibly due to the fact that 90% of them were treated by surgery. The data appeared to be in agreement with the results of a previous analysis based on the National Cancer Database in the US, which reported a 5-year OS rate of 89.9% in cases of broncho-pulmonary AdCC and 88.2% in cases of broncho-pulmonary MEC [14]. In contrast, a previous review article reported a 5-year OS rate of surgically treated broncho-pulmonary SGTCs of only 64.3% [17], which is appears inferior to our results in the present study.

This study had some limitations. Firstly, this was a retrospective study conducted based on data collected at a single institution, with a relatively small sample size, since the disease is rather rare, to allow arrival at any definitive conclusions. Secondly, even though the institution at which it was conducted is a large-volume general hospital, the number of patients who were referred and received the diagnosis might be influenced by possible activity difference according to departments. This might have resulted in biased patient enrollment for each organ site of involvement. Thirdly, the histological types of SGTCs in the enrolled patients were limited to SDC, AdCC, MEC, EMC, AcCC, and PAC, since the primary purpose of the study was to enable comparisons between SGTCs arising from the salivary glands and non-salivary gland sites. Fourthly, pathological diagnosis was given by several different pathologists without obtaining consensus. In addition, although the WHO histological classification was revised at 2017 [18] during the present study period, pathological reevaluation was not performed for patients diagnosed before the revision. Fifthly, a definitive comparison of the survival times was difficult because of the different stage distribution and different definitions of disease stages according to the primary site. Sixthly, patients referred to other institutions for treatment accounted for 11% (19/173) of the entire population. Although the main reason for this was advanced disease with poor performance status of the patients, uncertain information regarding this is likely to tell on the quality of the research. Despite these shortcomings, the present study, we believe, has clear advantages in terms of the novelty of determining the cross-organ demographics of SGTCs, which are rarely encountered tumors with unmet medical needs. In fact, we have clearly illustrated that SGTCs, which usually arise in the major and minor salivary glands can also arise from a variety of other organs. The distribution of the histological types varied among primary sites, which could explain, at least in part, the different prognoses according to the primary sites.

In conclusion, SGTCs, whether originating from the salivary glands or non-salivary gland organs, hold distinct clinical significance. A comprehensive understanding of these tumors arising across various organs is crucial for developing novel therapies for this group of tumors. Promising advances in molecular-targeted therapies and immunotherapy are currently in progress, offering potential avenues for further development.
